# Randomness and Statistical Laws of Indentation-Induced Pop-Out in Single Crystal Silicon

**DOI:** 10.3390/ma6041496

**Published:** 2013-04-12

**Authors:** Hu Huang, Hongwei Zhao, Chengli Shi, Lin Zhang, Shunguang Wan, Chunyang Geng

**Affiliations:** College of Mechanical Science & Engineering, Jilin University, Renmin Street 5988, Changchun 130025, China; E-Mails: huanghuzy@163.com (H.H.); shichengli163@163.com (C.S.); zhanglin1771@126.com (L.Z.); wanshunguang@126.com (S.W.); gengcy_career@yahoo.cn (C.G.)

**Keywords:** pop-out, single crystal silicon, maximum penetration load, loading/unloading rate, statistic

## Abstract

Randomness and discreteness for appearance of pop-out of the single crystal silicon with a (100) orientation were studied by a self-made indentation device. For a given maximum penetration load, the load *P*_po_ for appearance of pop-out fluctuates in a relatively large range, which makes it hard to study the effect of the loading/unloading rate on the load *P*_po_. Experimental results with different maximum penetration loads indicate that the critical penetration load for appearance of pop-out is in the range of 15 mN~20 mN for the current used single crystal silicon. For a given maximum penetration load, the load *P*_po_ for appearance of pop-out seems random and discrete, but in the point of statistics, it has an obviously increasing trend with increase of the maximum penetration load and also the fraction *P*_po_/*P*_max_ approximately keeps in the range of 0.2~0.5 for different maximum penetration loads changing from 15 mN to 150 mN.

## 1. Introduction

Because of excellent semiconducting properties, single crystal silicon (Si) has been widely used in microelectronics and optoelectronics. Research on single crystal silicon under external loads has been given much attention [1,2,3,4,5]. Nanoindentation as a powerful method to characterize properties of materials in micro/nano scale also has been used to study mechanical response of single crystal silicon under the penetration load [6,7,8,9,10,11,12,13,14,15,16]. 

Unlike general materials which have smooth load-depth curves, discontinuities in the unloading curves named pop-out and elbow were observed during indentation testing of single crystal silicon [7,10,12,13,14,15,16], which responded to phase transformations of silicon beneath the indenter. Results of Raman microspectroscopy analysis of nanoindentations indicate that pop-out corresponds to the formation of Si–XII and Si–III phases [17], and elbow resulted from the amorphization of silicon on pressure release [13,14,16,17].

Phase transformations of silicon are affected by many factors, such as indenter shapes [7,12], loading/unloading rates [12,18], cyclic loading conditions [2,16,19], maximum penetration loads [12,13,14,17], temperature [15] and so on [20]. Usually, pop-out appears easily when lower loading/unloading rates and higher maximum penetration loads are selected, while elbow may be observed more often when faster rates and lower maximum penetration loads are selected [17]. Yan *et al*. [14] reported that a small load (~20 mN) formed a complete amorphous indentation after unloading but a big load (~50 mN) produced a mixture of the amorphous and nano-crystalline structure beneath the indentation, and the critical load for this transition from the complete amorphous structure to the mixture of amorphous and nano-crystalline structure was approximately 30 mN. Lee *et al*. [13] reported the critical load for appearance of pop-out was around 40 mN for the device grade p-type single-crystal silicon wafers with a (100) orientation by indentation tests with three maximum penetration loads 30 mN, 40 mN and 70 mN, but the conclusion may be inaccurate because of very limited data points. Up to now, randomness and discreteness for appearance of pop-out as well as their statistical laws have not been studied. Also, the critical penetration load for the appearance of pop-out has not been revealed. Research on randomness and statistical laws of indentation-induced pop-out in single crystal silicon may be useful to understand the nature and influencing factors of phase transformations. In addition, research on the critical load is meaningful for selection of machining parameters of single crystal silicon because the mechanical contact conditions of the indentation test and the micro/nano machining processes, for example single point diamond machining, are similar [14]. 

In this paper, we study randomness and discreteness for appearance of pop-out as well as their statistical laws, and further research on the relationship between the maximum penetration load and appearance of pop-out of single crystal silicon with a (100) orientation by a self-made indentation device.

## 2. Materials and Experiments

**Figure 1:**
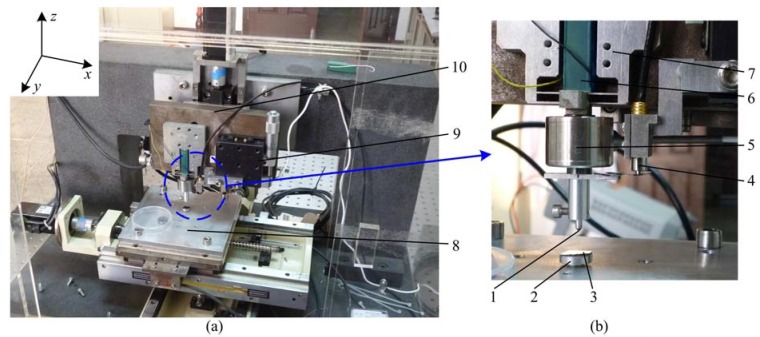
Photography of the self-made indentation device.

In this paper, a self-made indentation device was developed to carry out indentation experiments. Figure 1a is the prototype and Figure 1b is the local enlarge drawing of Figure 1a illustrating details of key components. The principle of the developed indentation device is similar to that in reference [21]. So, we only give a short description of the device here. The *x*-*y* positioning stage is used to realize positioning of the specimen. Macro-adjusting of the indenter in the *z*-axis is realized via the servo motor, and the fine penetration and withdraw of the indenter are realized via the piezoelectric actuator and the flexure hinge. Penetration depth and load can be measured by the displacement sensor (Micro-Epsilon, capaNCDT 6500) and the load sensor (Honeywell, Model 31 low) respectively. The aided-adjusting mechanism can be used to adjust the displacement sensor into its measuring range. Compared with the device in reference [21], layout of the displacement sensor and the load sensor in Figure 1b is different, which can increase the instrument stiffness with consideration of the compliance of the load sensor. 

After calibration, repeatability tests on fused quartz from Hysitron Inc. were carried out using a Berkovich indenter (Synton-MDP Ltd, Switzerland) with the face angle of 65.3° and the radius of curvature about 100 nm to verify feasibility of the indentation device. Then, indentation tests of single crystal silicon were carried out by the self-made indentation device to study randomness and statistical laws of indentation-induced pop-out in single crystal silicon.

## 3. Results and Discussion

**Figure 2:**
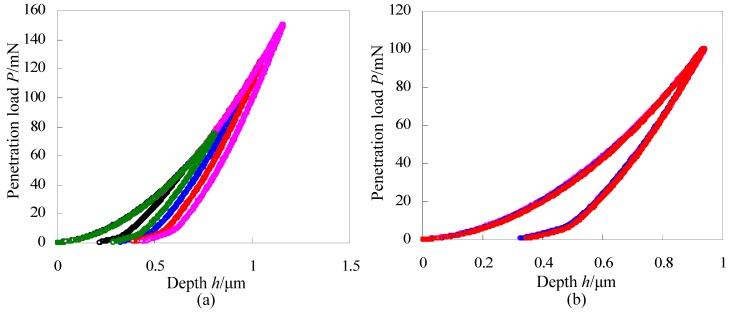
Repeatability tests on fused quartz with (**a**) different maximum penetration loads and (**b**) the same maximum penetration loads.

Comparing the load-depth curves with different maximum loads is a good method to evaluate the repeatability of the indentation instrument. Figure 2a gives load-depth curves of fused quartz with loads of 50 mN, 75 mN, 100 mN, 125 mN and 150 mN respectively. The loading curves agree well with each other and the unloading curves distinguish with each other because of different maximum penetration loads. Figure 2b gives load-depth curves of fused quartz with the same maximum penetration loads of 100 mN and these three curves agree well with each other. Figure 2a,b indicate that the developed indentation device has good repeatability. Hardness and elastic modulus of fused quartz are obtained from Figure 2b according to the Oliver-Pharr method [22], and they are 9.33 GPa and 70.1 GPa respectively which identify well with the data (*i.e.*, 9.25 GPa ± 10% and 69.6 GPa ± 5% for hardness and reduced modulus, respectively) provided by the Hysitron Inc. Results mentioned above indicate the feasibility of the developed indentation device. 

**Figure 3:**
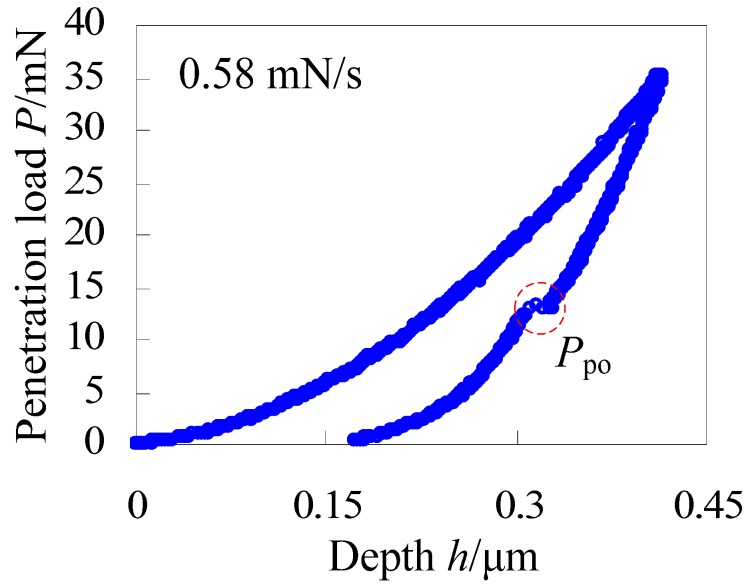
The load-depth curve of single crystal silicon with the maximum penetration load of 35 mN and the loading/unloading rate of 0.58 mN/s.

Via the calibrated indentation device, indentation experiments of single crystal silicon were carried out. Figure 3 is the load-depth curve of single crystal silicon with the maximum penetration load of 35 mN and the loading/unloading rate of 0.58 mN/s. Under this condition, obvious pop-out is observed during the unloading portion. According to the Oliver-Pharr method [22], hardness and elastic modulus of the used single crystal silicon are also obtained, and they are 12.84 GPa and 178.6 GPa respectively which agree well with the measured values, 12.62 GPa and 176.1 GPa by the CSM’s Nanoindentation Tester. This further verifies feasibility of the indentation device. 

As mentioned in references [12,14,17,18], loading/unloading rates and maximum penetration loads affect occurrence of pop-out. Here, we focus on whether the load *P*_po_ for appearance of pop-out for a given maximum penetration load is a constant and whether the loading/unloading rate and maximum penetration loads affect the load *P*_po_ for appearance of pop-out. 

**Figure 4:**
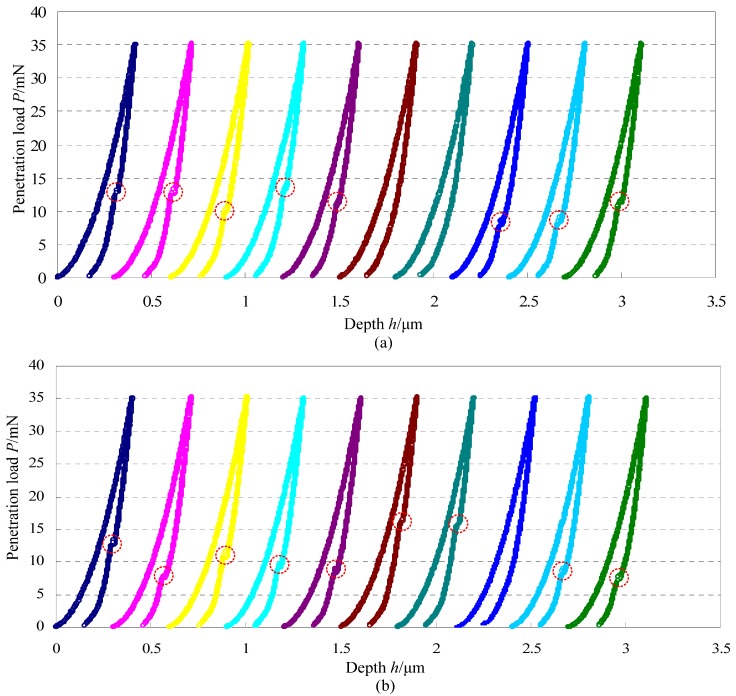
Load-depth curves of single crystal silicon with the same maximum penetration load of 35 mN and the same loading/unloading rate of 0.58 mN/s.

**Figure 5:**
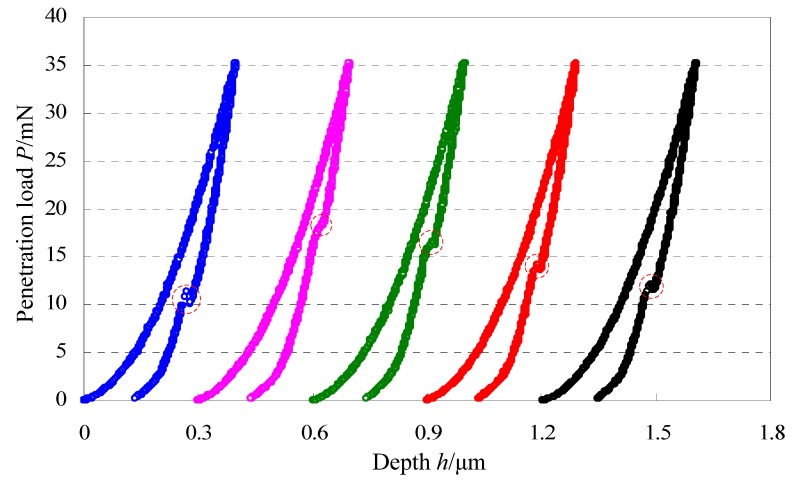
Load-depth curves of single crystal silicon with the same maximum penetration load of 35 mN and the same loading/unloading rate of 0.70 mN/s.

Figure 4 gives 20 load-depth curves of single crystal silicon with the same maximum penetration load of 35 mN and the same loading/unloading rate of 0.58 mN/s. As shown in Figure 4a,b, the position for appearance of pop-out for each curve is marked by the circle. During the 20 indentation experiments, pop-out appears 17 times and there are 3 times that pop-out does not appear during the unloading portion. Obviously, the load *P*_po_ for appearance of pop-out is not a constant and it changes in a relatively large range of 5~15 mN. Because of the wide range of the load *P*_po_ for appearance of pop-out with the same maximum penetration load and the same loading/unloading rate, it is hard to study the effect of the loading/unloading rate on the load *P*_po_. In order to further address this issue, five indentation tests of single crystal silicon were carried out with the same maximum penetration load of 35 mN and the same loading/unloading rate of 0.70 mN/s, and results are illustrated in Figure 5. Just like Figure 4, the load *P*_po_ for appearance of pop-out also has large fluctuations. By combination of Figure 4 and Figure 5, randomness for appearance of pop-out mixes the effect of the loading/unloading rate on the load *P*_po_ for appearance of pop-out. However, the reasons leading to this randomness are not clear and need more further study in the future. 

**Figure 6:**
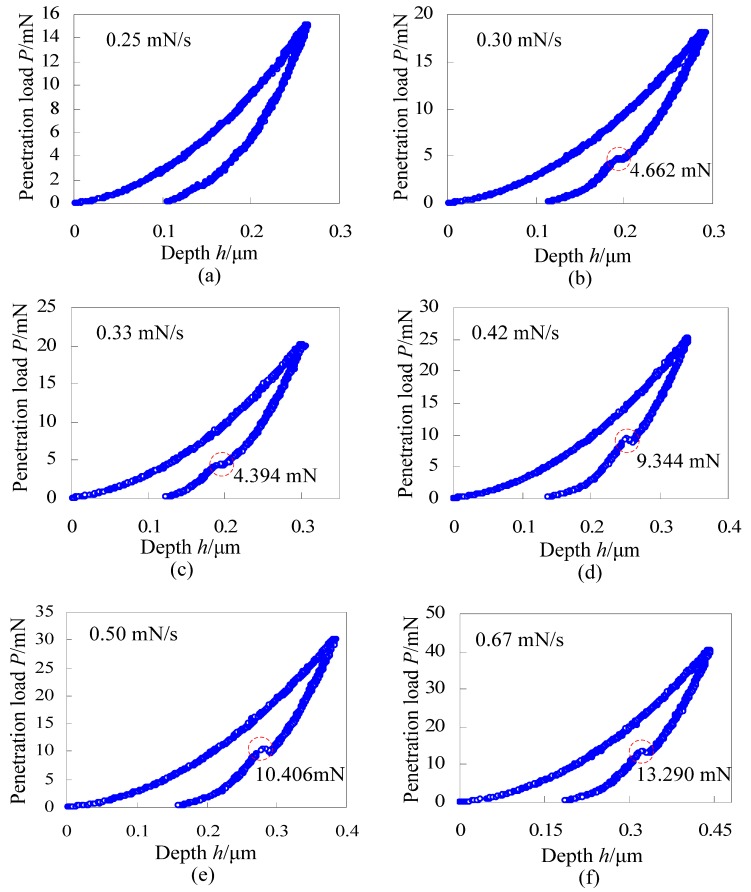
Load-depth curves of single crystal silicon with different maximum penetration loads and the same testing time of 100 s.

Next, we will consider the effect of maximum penetration loads on the load *P*_po_ for appearance of pop-out, ignoring the effect of the loading/unloading rate. Indentation tests of single crystal silicon with different maximum penetration loads and the same testing time of 100 s were carried out. In order to save time, following indentation tests were all set to be 100 s. Load-depth curves of single crystal silicon with the maximum penetration loads of 15 mN, 18 mN, 20 mN, 25 mN, 30 mN and 40 mN are illustrated in Figure 6a–f, respectively. When the maximum penetration load is 15 mN, there is no obvious pop-out, as shown in Figure 6a. However, when maximum penetration loads are 18 mN, 20 mN, 25 mN, 30 mN and 40 mN, obvious pop-out appears. So, the critical penetration load for appearance of pop-out is in the range of 15~20 mN for the current used single crystal silicon, which is less than values reported by Yan *et al*. (~30 mN) and Lee *et al*. (~40 mN). Moreover, the load *P*_po_ for appearance of pop-out approximatively increases with increase of the maximum penetration loads for these random indentation tests with maximum penetration loads of 18 mN, 20 mN, 25 mN, 30 mN and 40 mN. 

**Figure 7:**
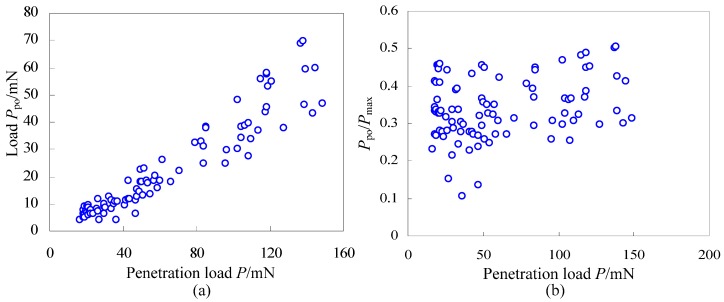
(**a**) The load *P*_po_ for appearance of pop-out *versus* the maximum penetration load and (**b**) *P*_po_/*P*_max_ *versus* the penetration load.

As shown in Figure 4, the load *P*_po_ for appearance of pop-out with the same maximum penetration load and the same loading/unloading rate changes in a relatively large range, and it seems random and irregular. However, the load *P*_po_ for appearance of pop-out approximatively increases with increase of the maximum penetration loads in Figure 5. So, the question is whether there is a law for the effect of maximum penetration loads on the load *P*_po_ for appearance of pop-out. In order to find this potential law, 100 indentation tests of single crystal silicon with different maximum penetration loads from 15 mN to 150 mN were carried out and there are 95 times that the pop-out appears. In terms of statistics, the relationship curve between the load *P*_po_ for appearance of pop-out and the maximum penetration load is illustrated in Figure 7a, and the relationship curve between the fraction *P*_po_/*P*_max_ (Divide the load *P*_po_ by the maximum penetration load) and the maximum penetration load is illustrated in Figure 7b. Though the load *P*_po_ for appearance of pop-out with the same maximum penetration load and the same loading/unloading rate seems random and irregular, it has an obviously increasing trend with increase of the maximum penetration loads as shown in Figure 7a, which means the higher the maximum penetration load is, the higher the load *P*_po_ for appearance of pop-out may be. On the other hand, the fraction *P*_po_/*P*_max_ approximately keeps in the range of 0.2~0.5 though these data points are discrete distribution in Figure 7b. In summary, for a given maximum penetration load, the load *P*_po_ for appearance of pop-out seems random and discrete, but in the point of statistics, it has an obviously increasing trend with increase of the maximum penetration loads and also the fraction *P*_po_/*P*_max_ approximately keeps in the range of 0.2~0.5 for different maximum penetration loads changing from 15 mN to 150 mN.

## 4. Conclusion

In summary, a self-made indentation device was developed to study pop-out phenomenon of single crystal silicon with a (100) orientation. Repeatability tests on fused quartz indicate that the indentation device has good repeatability, and simulation results of hardness and elastic modulus by the Oliver-Pharr method verify the testing accuracy. Then, indentation experiments of single crystal silicon were carried out via the calibrated indentation device. Experimental results suggest that for a given maximum penetration load and the loading/unloading rate, the load *P*_po_ for appearance of pop-out fluctuates in a relatively large range, which makes it hard to study the effect of the loading/unloading rate on the load *P*_po_. Experiments with changed loading/unloading rates further address this issue. Experimental results with different maximum penetration loads indicate that the critical penetration load for appearance of pop-out is in the range of 15~20 mN for the current used single crystal silicon, which is less than values reported by Yan *et al*. (~30 mN) and Lee *et al*. (~40 mN). The maximum penetration loads change the load *P*_po_ for appearance of pop-out. Larger loads increase the formation of nanocrystalline phase [13] and lead to brittle damage of single crystal silicon, while small loads cause the amorphous structure which is benefit for ductile machining of single crystal silicon. So the ideal normal load for single point diamond machining of single crystal silicon with a (100) orientation should be constrained to be less than 15 mN. Though the load *P*_po_ for appearance of pop-out seems random and discrete for a given maximum penetration load, it has an obviously increasing trend with increase of the maximum penetration loads. The fraction *P*_po_/*P*_max_ approximately keeps in the range of 0.2~0.5 for different maximum penetration loads changing from 15 mN to 150 mN in the point of statistics. 
